# Integrated management of multidrug-resistant tuberculosis, HIV, and hepatitis B co-infection in Ghana: a case report

**DOI:** 10.3389/fmed.2026.1801109

**Published:** 2026-06-15

**Authors:** Stanley Yaidoo, Judith Maame Tanoa Yelbert, Rodger MacScott-Lutterodt, Elizabeth Nkumah Obeng, Stephannie Kafui Amenya

**Affiliations:** 1 Awutu Senya East Health Directorate, Ghana Health Service, Kasoa, Ghana; 2 University of Ghana Medical Centre, Legon, Ghana; 3 Greater Accra Regional Hospital, Accra, Ghana

**Keywords:** case report, co-infection, hepatitis B, HIV, multidrug- resistant tuberculosis, renal failure, rifampicin-resistant

## Abstract

**Introduction:**

Co-infection with multidrug-resistant tuberculosis (MDR-TB) and human immunodeficiency virus (HIV) represents a major global public health challenge. The coexistence of these conditions with chronic hepatitis B infection further complicates clinical management due to overlapping drug toxicities, limited treatment options, and challenges with patient adherence, particularly in resource-constrained settings.

**Case presentation and clinical findings:**

A 37-year-old woman living with HIV presented to a clinic in Ghana’s Central Region with symptoms suggestive of pulmonary tuberculosis (TB). Further evaluation led to the diagnosis of rifampicin-resistant pulmonary TB, chronic hepatitis B infection, and chronic renal impairment. Her clinical course was complicated by adverse drug reactions, chronic renal disease and intermittent interruptions in antiretroviral therapy due to drug stockouts. These factors posed significant challenges to treatment continuity and tolerability.

**Conclusion:**

Despite significant clinical and systemic challenges, the patient completed a 12-month MDR-TB regimen with documented microbiological cure. Antiretroviral therapy for HIV is ongoing, while the hepatitis B infection and chronic renal disease are under clinical monitoring without specific antiviral treatment or dialysis due to cost constraints. This case highlights the critical importance of integrated, multidisciplinary care in the management of complex co-infections. It also draws attention to persistent health system constraints, including drug shortages, limited diagnostic capabilities, and financial barriers to accessing healthcare, which must be addressed to improve outcomes for patients with similar high-risk profiles in low-resource settings.

## Introduction

Tuberculosis (TB) and human immunodeficiency virus (HIV) infection remain significant global public health concerns despite the availability of existing prevention and control strategies (1). The intersection of multidrug-resistant tuberculosis (MDR-TB), HIV, and hepatitis B virus (HBV) infection presents one of the most complex challenges in infectious disease management, particularly in resource-limited settings. The co-existence of these multiple infections in an individual complicates diagnosis, treatment, and prognosis, placing a substantial burden on healthcare systems and patients alike (2).

In 2023, an estimated 400,000 people developed rifampicin-resistant or MDR-TB (MDR/RR-TB), resulting in approximately 150,000 deaths globally (2). Although the World Health Organization (WHO) African Region has experienced a gradual decline in MDR/RR-TB incidence since 2015, 3.2% of new TB cases and 16% of previously treated cases still involved drug resistance as of 2023 (2).

In Ghana, the epidemiological context is particularly concerning. Approximately 13.6% of HIV-positive individuals are co-infected with HBV; thus, approximately one in seven HIV patients is chronically infected with HBV (3). TB/HIV co-infection rates range from 10.9% to 22.9% across different regions, with a prevalence of 10.92% reported in the Central Region (4, 5). HIV infection not only increases the risk of developing active TB by 26–31 times but is also associated with increased MDR-TB incidence and poorer treatment outcomes (6–8).

Despite WHO recommendations, routine HBV screening among HIV-positive patients is not widely implemented in Ghana (3). The cost of managing chronic HBV infection, estimated at $100–150 per month, places treatment beyond the financial reach of most patients, making vaccination the most viable preventive strategy (3). Furthermore, TB/HIV co-infection reduces treatment success rates from 91.2% in HIV-negative patients to 77% among those with HIV (5).

## Case presentation

In October 2022, a 37-year-old woman with a known history of HIV infection receiving antiretroviral therapy (ART) presented to a public health facility in the Awutu Senya East Municipality with a 1-month history of persistent productive cough, low-grade fever, night sweats, weight loss, and fatigue. Following her HIV diagnosis, the patient began an ART regimen in October 2021. At the time of initiation, her baseline GeneXpert test for pulmonary TB was negative. However, following the onset of new respiratory and constitutional symptoms in October 2022, repeat sputum GeneXpert tests detected *Mycobacterium tuberculosis* with rifampicin resistance. There was no history of contact with individuals with chronic cough. She denied intravenous drug use or prior blood transfusion but reported a history of unprotected sexual exposure. Before presentation, she was not taking any medications other than ART.

The patient was a market vendor and widowed. Menstrual history was unremarkable, and she was not pregnant at presentation. She had received childhood immunizations, including Bacille Calmette-Guérin (BCG). There was no family history of TB, renal disease, or liver disease.

### Clinical investigations and findings

Upon presentation to the facility in October 2022, the 37-year-old woman complained of a persistent cough, generalized body weakness, intermittent fever, and significant weight loss over the preceding month. Her vital signs were as follows: blood pressure of 130/96 mmHg, pulse rate of 86 beats/min, temperature of 36.2 °C, and a random blood glucose level of 5.1 mmol/L. At the time of her visit, she weighed 67 kg.

The physical examination was unremarkable, with the exception of bilateral coarse crackles noted during lung auscultation. No signs of lymphadenopathy, jaundice, or hepatosplenomegaly were observed.

**Figure 1:**
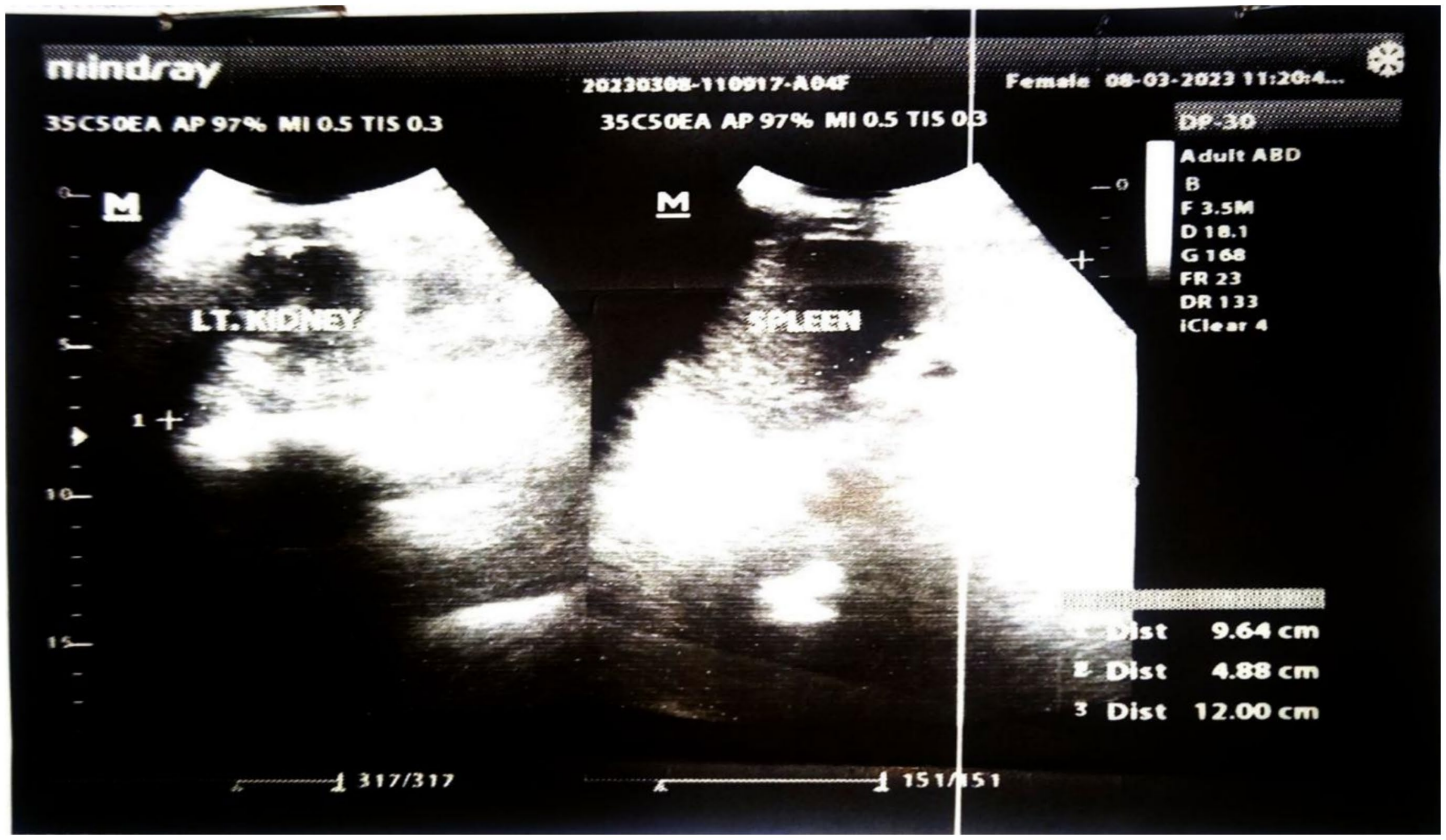
An abdominal ultrasound showing normal-sized kidneys with echogenic cortices and a loss of corticomedullary differentiation (August 2023).

**Figure 2:**
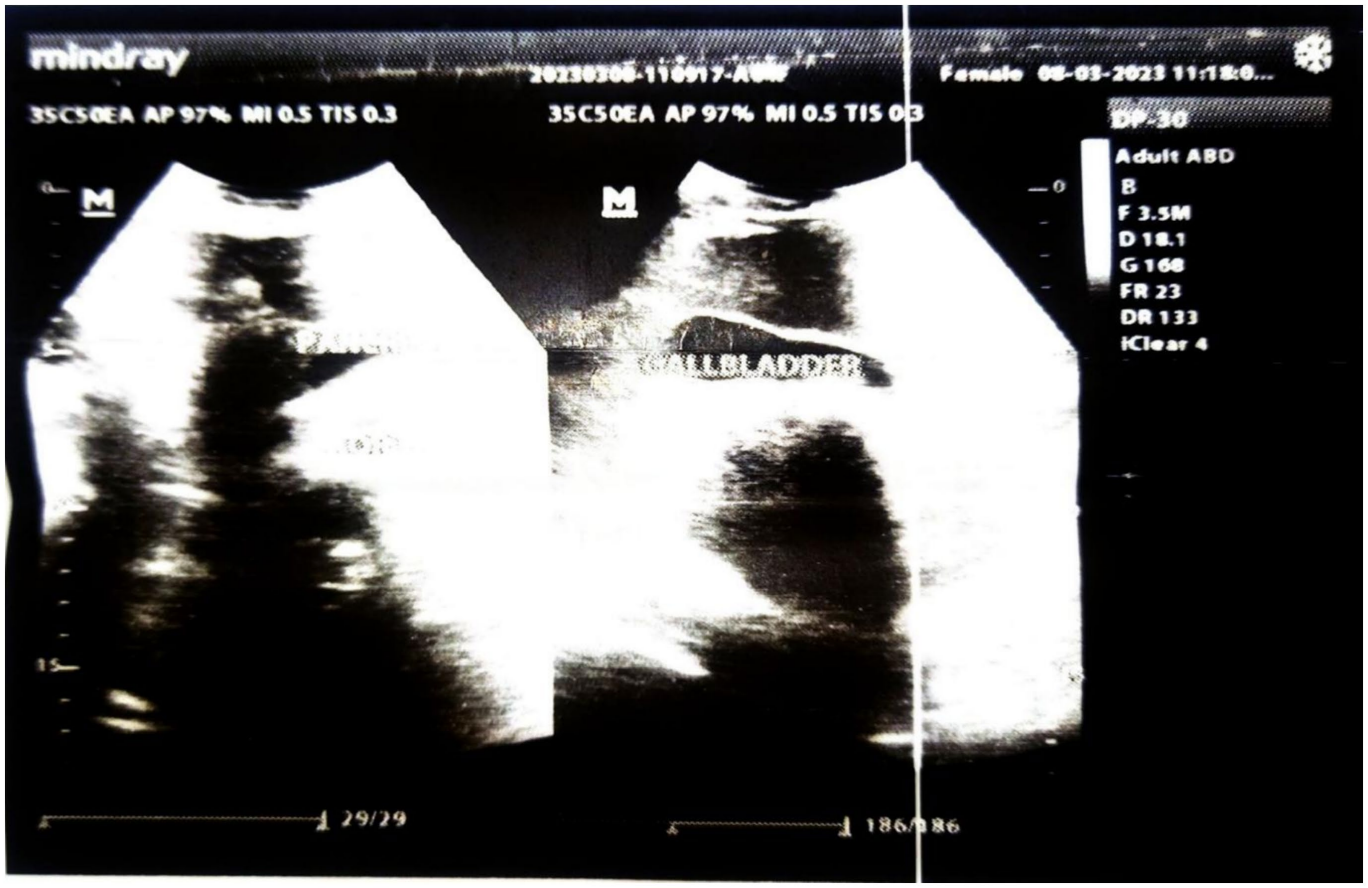
An ultrasound scan showing a normal pancreas and gallbladder (August 2023).

**Figure 3:**
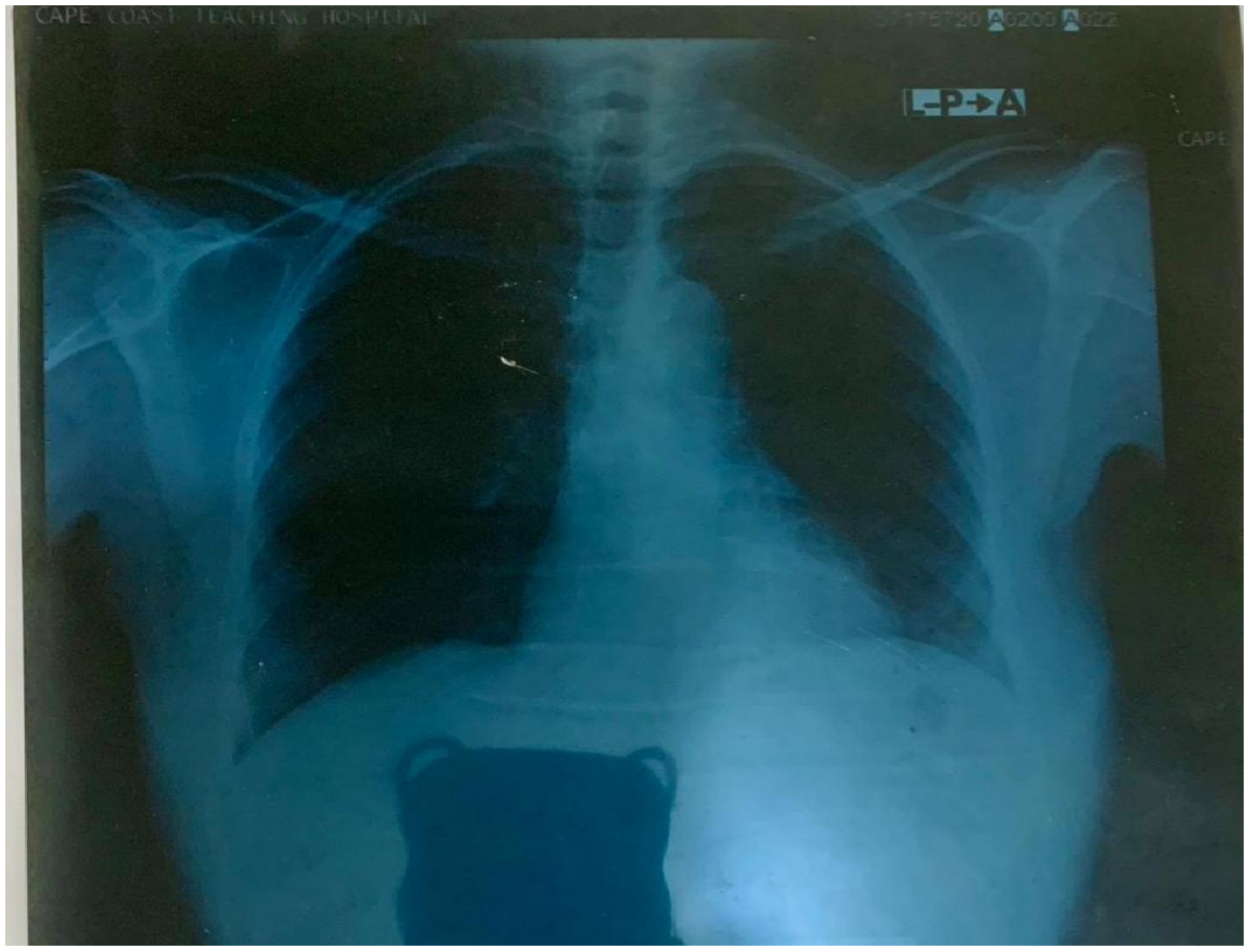
Chest radiograph (posteroanterior view), obtained on 28 October 2022, demonstrating no obvious focal consolidation; the findings were subtle and interpreted in the context of clinical findings and microbiologically confirmed MDR-TB.

**Figure 4:**
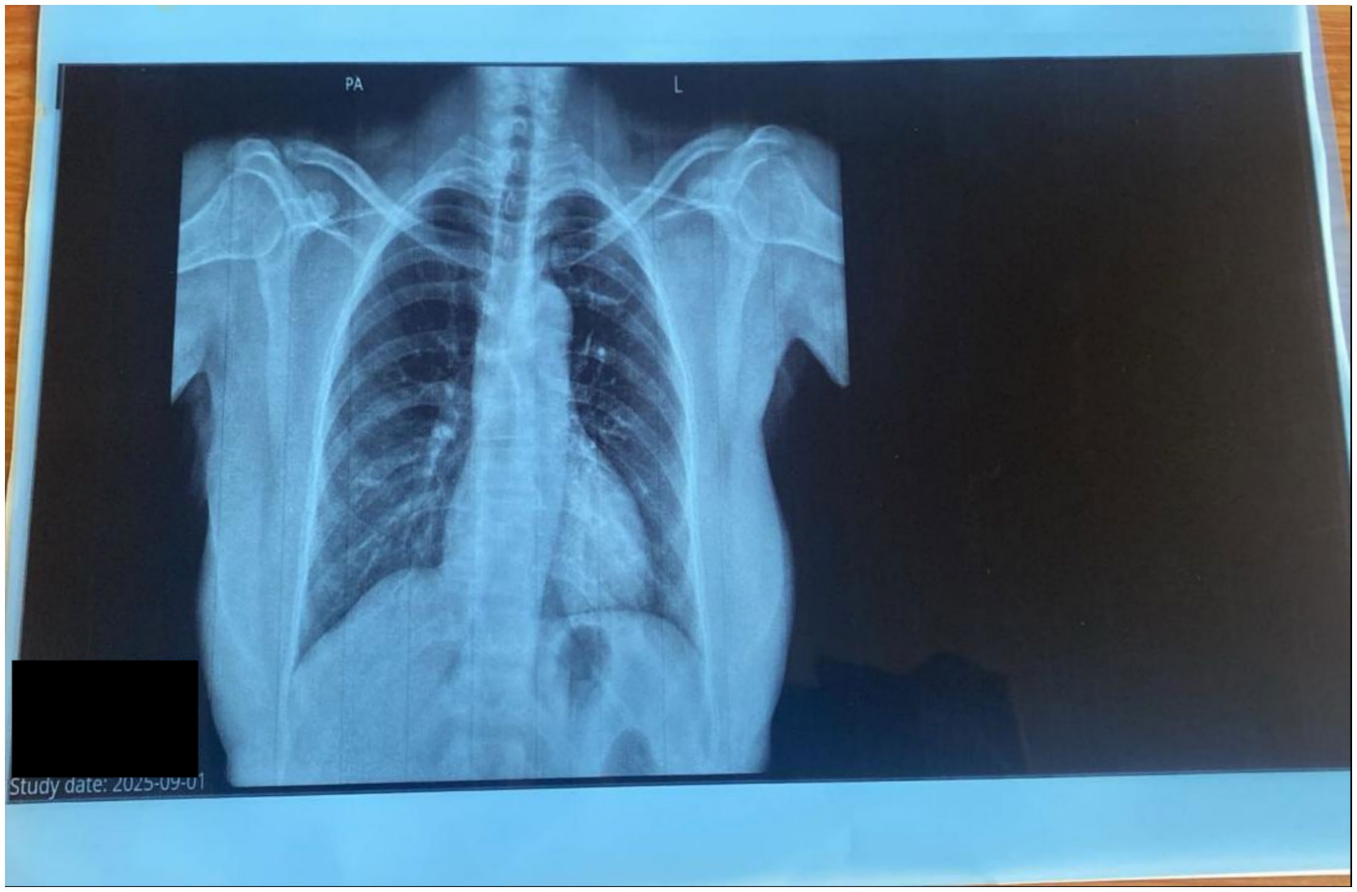
Chest radiograph obtained on 1 September 2025, showing a thin-walled cavity in the left upper lung zone in a patient previously treated for tuberculosis.

Key laboratory and imaging findings included the following:

- Sputum microscopy and GeneXpert: These tests were positive for *Mycobacterium tuberculosis* with rifampicin resistance.
- HIV rapid antibody test: This test returned a positive result.
- Chest X-ray: Chest radiograph (posteroanterior view) demonstrated no obvious focal consolidation; findings were subtle and interpreted in the context of clinical symptoms and microbiologically confirmed MDR-TB.
- Abdominal ultrasound: Kidneys were bilaterally normal in size but hyperechogenic, with poor corticomedullary differentiation suggestive of chronic bilateral renal impairment. All other visualized organs were unremarkable.
- Liver function test: The results of the liver function test were normal at the time of initiation. In April 2026, albumin was 44.2 N, Alanine Aminotransferase (ALT) was 23.8 N, gamma-glutamyl transferase was 25.9 N, and ALP was 193.5 H.
- Hepatitis B profile: Hepatitis B surface antigen and core antibody were positive; all other parameters were non-reactive.
- Complete blood count: Hemoglobin is 7.1 g/dL; white blood cell count is 3.5 × 10^9^/L; platelet count is 133 × 10^9^/L.
- CD4 count was not performed in accordance with prevailing national guidelines.
- HIV viral load at 6 months after ART initiation was 50.1 copies/mL, with a subsequent result of “target not detected.” The most recent viral load in April 2026 also showed “target not detected.”
- HBV DNA testing was not performed due to financial constraints, as hepatitis B investigations and treatment are not covered under national programs. Serologic testing demonstrated hepatitis B surface antigen and core antibody positivity, suggestive of a late phase of infection with low or non-replicative activity.
- Baseline renal function before MDR-TB treatment showed impairment (creatinine, 173 μmol/L; urea, 6.2 mmol/L; estimated glomerular filtration rate [eGFR], 52.7 mL/min/1.73 m^2^). Progressive deterioration was observed during follow-up (creatinine up to 543.3 μmol/L; urea, 9.5 mmol/L; eGFR, 8 mL/min/1.73 m^2^) in April 2026 (see Figures 1–4).

### Diagnostic assessment

Initial screening for TB was conducted using sputum smear microscopy, followed by GeneXpert MTB/RIF, which confirmed *Mycobacterium tuberculosis* with rifampicin resistance, establishing a diagnosis of MDR-TB. Hepatitis B profile testing revealed hepatitis B surface antigen and core antibody positivity (see Table 1).

**Table 1:** Chronology of events.

Time period	Clinical events/findings	Treatment/management	Diagnostic tests performed
Pre-October 2022	On ART for 1 year Presumed hepatitis B infections	Initiated tenofovir-based ART regimen	1. First Response HIV-1/2 (screening) 2. Oraquick HIV-1/2 3. SD Bioline (confirmatory) 4. Kidney function test 5. Liver function test 6. Hepatitis B surface antigen rapid test 7. HIV viral load
October 2022 (initial diagnosis)	Diagnosed with: Rifampicin-resistant TB Hepatitis B infection	Initiated second-line TB treatment Pyridoxine prophylaxis	1. Sputum GeneXpert 2. Chest X-ray 3. Hepatitis B surface antigen rapid test 4. Hepatitis B serology panel
Month 3 review	Suspected linezolid-induced anemia and leucopenia Reduced cough Weight: 62.5 kg	Continued TB treatment and monitoring	1. Full blood count 2. Blood film comment 3. Sputum microscopy for *Mycobacterium tuberculosis*
Month 5 review	Clinically stable Numbness in feet	TDF discontinued Switched to ABC	1. Liver function test 2. Kidney function test 3. Full blood count
Month 8 review	Enrolled at the renal clinic at Cape Coast Teaching Hospital	Continued ART and renal follow-up	1. Liver function test 2. Kidney function test
Month 11 review	ART stockout	Continued anti-TB therapy ART stockout for 1 month	
Post-treatment review	Negative sputum smear No growth on TB culture	Continued ART	Sputum microscopy for *Mycobacterium tuberculosis*

ABC, abacavir; ART, antiretroviral therapy; HIV, human immunodeficiency virus; TB, tuberculosis; TDF, tenofovir disoproxil fumarate.

Chest radiography showed no overt focal consolidation; the imaging findings were subtle and interpreted in conjunction with clinical chest findings, confirming an MDR-TB diagnosis. Abdominal ultrasound revealed hyperechogenic renal cortices, raising suspicion of chronic kidney injury (CKD), possibly related to HIV nephropathy, drug toxicity, or other systemic illness. The laboratory workup included renal function tests and hematologic studies, both essential for monitoring drug safety, particularly for tenofovir disoproxil fumarate (TDF) and linezolid.

Key diagnostic limitations included the lack of access to second-line drug susceptibility testing (DST) for agents such as linezolid and bedaquiline, which hindered individualized resistance-guided therapy as well as the unavailability of HBV DNA testing. Additionally, immune reconstitution inflammatory syndrome was considered a potential concern following re-initiation of ART.

Negative prognostic factors included the following:

- Multiple co-infections with MDR-TB, HIV, and HBV, increasing the risks of mortality, drug interactions, and treatment complexity.
- Drug-induced toxicities, notably TDF-associated nephropathy and linezolid-induced leukopenia.
- ART stockouts leading to treatment interruptions.

Positive prognostic indicators that contributed to a favorable outcome include the following:

- High treatment adherence.
- Timely adjustment of ART from TDF-based therapy to abacavir (ABC)-based therapy to mitigate renal injury.

### Therapeutic intervention

Following the confirmation of rifampicin resistance, the patient was initiated on a second-line MDR-TB regimen that included ethambutol, levofloxacin, clofazimine, pyrazinamide, linezolid, bedaquiline, and pyridoxine.

Her ART regimen initially consisted of a fixed-dose combination of TDF, lamivudine (3TC), and dolutegravir (DTG). After 3 months, she reported a reduced cough but developed progressive weight loss, gastritis, anemia, and leukopenia. These findings were attributed to linezolid-induced myelosuppression, prompting the discontinuation of linezolid.

By the fifth month of treatment, the patient developed peripheral neuropathy. Furthermore, CKD was considered likely to have been aggravated by TDF; therefore, her ART regimen was changed to ABC, 3TC, and DTG. Subsequent HIV viral load monitoring following the regimen change demonstrated sustained virological suppression, with the most recent result showing “target not detected.” She was subsequently enrolled at the renal clinic at Cape Coast Teaching Hospital for further management of CKD.

In the eleventh month, stockout of ABC/3TC/DTG resulted in the interruption of ART, whereas anti-TB treatment continued without interruption. No additional therapeutic agents were introduced during this period. Hematinics were prescribed to address persistent anemia.

### Follow-up and outcomes

The patient completed a 12-month course of MDR-TB treatment on 27 October 2023, demonstrating post-treatment sputum smear negativity and no growth on culture, consistent with microbiological cure. Clinically, she showed sustained improvement, including increased energy levels, improved appetite, and weight gain.

She remains on ART and hematinic support, with good adherence following earlier interruptions. After switching to the ABC-based ART regimen, HIV viral load monitoring demonstrated sustained virological suppression, with the most recent result in February 2026 showing “target not detected.” Hematological indices improved, with a hemoglobin level of 11.0 g/dL and a white blood cell count of 5.82 × 10^9^/L recorded on 1 September 2025.

Renal function progressively declined during the course of management, with an eGFR of 8 mL/min/1.73 m^2^ in April 2026, consistent with end-stage kidney disease. Although the patient was referred multiple times for renal replacement therapy, she has been unable to access dialysis because of financial constraints. She continues to receive multidisciplinary care, including follow-up at both HIV and renal clinics.

## Discussion

This case highlights the clinical complexity of managing multiple infections, including MDR-TB, HIV, and chronic HBV in a resource-limited setting. While TB/HIV co-infection is common in Ghana, the presence of chronic HBV infection further complicates treatment due to overlapping toxicities and limited therapeutic options.

The patient was treated with an all-oral MDR-TB regimen including linezolid, in accordance with WHO recommendations (9). However, linezolid-induced leukopenia necessitated the discontinuation of the drug, reflecting the well-documented hematologic toxicity associated with prolonged use. The WHO consolidated guidelines for drug-resistant TB emphasize the importance of close safety monitoring during linezolid therapy, including regular full blood count assessment for early detection of myelosuppression and clinical monitoring for peripheral neuropathy, with prompt dose adjustment or discontinuation where indicated (9).

Renal impairment was confirmed by ultrasound findings and serial biochemical monitoring, prompting a switch from a tenofovir disoproxil fumarate (TDF)-based ART regimen to an ABC-based regimen, in accordance with Department of Health and Human Services (DHHS) guidance for patients with impaired renal function (10).

HIV treatment outcomes were favorable, with viral suppression achieved (50.1 copies/mL at 6 months and subsequently undetectable), underscoring the effectiveness of ART despite the presence of comorbidities. CD4 monitoring was not performed because HIV treatment and monitoring in Ghana primarily focus on viral load testing. CD4 testing is currently restricted to assessing advanced HIV disease and is not routinely performed in all patients due to the ongoing scale-up of point-of-care testing in Ghana, which aligns with evolving national and global practices that prioritize viral load testing over routine immunological monitoring except in cases of advanced disease (11, 12).

HBV management remained suboptimal. Although TDF is active against both HIV and HBV, its discontinuation without substituting another HBV-active agent (e.g., entecavir) may increase the risk of HBV reactivation or disease progression (10). Following the regimen change, HBV infection was monitored using serial liver function tests and clinical assessments, as HBV DNA testing was not feasible due to financial constraints. Although alternative HBV-active therapy, such as entecavir, was considered, it was not initiated because of cost and limited access within the public health system. This reflects the real-world challenges in HBV management in resource-limited settings, where HBV care remains largely patient-funded despite its significant contribution to liver-related morbidity and mortality (11).

Renal disease significantly complicated management. The patient had baseline renal impairment, which progressed to advanced chronic kidney disease (CKD) during follow-up. Although causality could not be definitively established, potential contributors include HIV-associated nephropathy, drug-related nephrotoxicity, and systemic illness. Despite multiple referrals for renal replacement therapy, the patient was unable to access dialysis because of financial barriers, reflecting systemic limitations in access to specialized care in low-resource settings.

Limited access to comprehensive drug susceptibility testing (DST) also posed a challenge. Although GeneXpert confirmed rifampicin resistance, DST for second-line and newer agents such as bedaquiline was unavailable, which is a common limitation in low-resource settings (9). This limits the ability to individualize MDR-TB regimens and may negatively impact long-term outcomes.

Despite these significant clinical and systemic challenges, microbiological cure of pulmonary MDR-TB was achieved following a 12-month treatment course. This favorable outcome was likely supported by good adherence to the treatment, timely adjustments in therapy in response to adverse events, and a multidisciplinary care approach. The patient continues to receive ART and is being followed up at both HIV and renal clinics.

This case underscores the need for integrated, patient-centered care models and highlights critical gaps in HBV management, access to renal care, and diagnostic capacity in high-burden, resource-limited settings.

### Patient perspective

The patient stated that receiving a diagnosis of three concurrent infections was a frightening experience. She described the initial phase of treatment as particularly challenging, marked by significant weakness and nausea. However, she felt supported by the healthcare team, who thoroughly explained the treatment plan and provided supportive care, including vitamins to help manage side effects. Over time, she noticed gradual improvement in her condition, regaining her strength and resolving her cough. Reflecting on her experience, she expressed deep gratitude for the medical care she received and stated that, although the journey was difficult, she now feels healthy and grateful to be alive.

### Strengths

1. The report describes the co-existence of MDR-TB, HIV, chronic HBV infection, and advanced renal disease, an uncommon and highly challenging combination that is underrepresented in the literature.
2. Achieving microbiological cure in a patient with multiple comorbidities and systemic constraints provides valuable real-world evidence regarding the feasibility of MDR-TB management in complex cases.
3. This case highlights critical health system challenges, including limited access to HBV diagnostics, insufficient funding for HBV care, and barriers to renal replacement therapy. These issues are highly relevant in many low- and middle-income countries.
4. Clinical decisions (e.g., ART modification, MDR-TB regimen, and monitoring approach) were well aligned with WHO and Ghana national guidelines, enhancing the scientific credibility and policy relevance of the report.
5. The manuscript provides detailed follow-up data, including microbiological, virological, hematological, and renal outcomes over time, allowing a comprehensive assessment of treatment response and disease progression.
6. The case highlights the consequences of interrupted HBV-active therapy and limited access to HBV care, contributing to the underrepresented literature on HBV management in HIV co-infected patients in sub-Saharan Africa.
7. The report reflects coordinated management across TB, HIV, and renal services, illustrating both the importance and challenges of integrated care models for complex cases.
8. The findings provide actionable policy-related insights for strengthening health systems, including the need for expanded diagnostic capacity, financial risk protection, and integration of HBV services into existing HIV/TB programs.

## Limitations

1. As a single case report, the findings cannot be generalized to broader MDR-TB/HIV populations, in whom treatment responses and outcomes can vary widely.
2. There were incomplete baseline clinical data, including CD4 count and HBV DNA testing.
3. Limited access to second-line and newer anti-TB drug susceptibility testing was noted.
4. Lack of access to renal replacement therapy may have influenced clinical outcomes.
5. There is potential confounding in attributing renal deterioration to specific etiologies (e.g., HIV-associated nephropathy vs. drug toxicity).

## Conclusion

This case demonstrates the substantial clinical and health system challenges associated with managing MDR-TB in the context of HIV infection, chronic hepatitis B, and progressive renal impairment in a resource-limited setting. Despite incomplete baseline investigations, treatment-limiting toxicities, and limited access to HBV diagnostics and renal replacement therapy, the patient achieved microbiological cure of MDR-TB following a 12-month all-oral regimen, supported by good adherence, timely regimen modification, and multidisciplinary care. The sustained HIV viral suppression further demonstrates the effectiveness of adapted ART strategies in the setting of renal dysfunction. However, the absence of HBV DNA testing, inability to initiate HBV-directed therapy, and lack of access to dialysis highlight critical structural gaps in care. This case underscores the urgent need for integrated service delivery, expanded access to viral hepatitis diagnostics and treatment, financial risk protection for high-cost conditions, and strengthened laboratory capacity, including advanced TB drug susceptibility testing, to improve outcomes in patients with complex co-infections in low-resource settings.

## Data Availability

The original contributions presented in the study are included in the article/Supplementary material. Further inquiries can be directed to the corresponding author.
